# Supplementary material for: Garcinol Promotes the Formation of Slow-Twitch Muscle Fibers by Inhibiting p300-Dependent Acetylation of PGC-1α
Source: Int J Mol Sci. 2023 Jan 31;24(3):2702. doi: 10.3390/ijms24032702 (PMC9916769; doi:10.3390/ijms24032702)

Garcinol Promotes the Formation of Slow-Twitch Muscle Fibers by Inhibiting p300-Dependent Acetylation of PGC-1α

Online Supplementary Material

Supplemental Table S1

Ingredient and chemical composition of the diets

**Item**	
Ingredient, %	
Corn	67.0
Soybean meal, dehulled	14.0
Wheat bran	15.6
Fish meal	0.5
Soybean oil	-
Dicalcium phosphate	1.2
Limestone	0.8
L-Lysine HCl (78%)	-
Salt	0.38
Premix [1]	0.52
Total	100
Calculated composition	
Gross energy, GE, MJ/kg	14.21
Crude protein, CP, %	21.7
Fat,%	9.4
Ca, %	0.95
Total P, %	0.61
Analyzed composition	
Metabolizable energy, ME, MJ/kg	12.04
Crude protein, CP, %	20.5
Fat,%	7.6
Ash, %	5.4
Ether extract, EE, %	1.7

[1]Premix supplied the following per kilogram of diet: 1) cobalt (as cyanocobalamin), 0.6; 2) copper (as $CuSO_4$ -$5H_2O$), 5.0; 3) iodine (as CaI_2), 0.48; 4) iron (as $FeSO_4$), 75.0; 5) manganese (as MnO), 20.0; 6) selenium (as Na_2SeO_3), 0.40; and 7) zinc (as ZnO), 10.0; 8) all-rac-a-tocopheryl acetate, 64.0; 9) D -biotin, 0.2; 10) calcium

d-pantothenate, 24.0; 11) cholecalciferol, 5.5; 12) folic acid, 6.0; 13) menadione sodium bisulfite, 2.2; 14) nicotinic acid, 30.3; 15) pyridoxine-HCl, 12.0; 16) retiny acetate, 1.9; 17) riboflavin, 5.5; 18) thiamin-HCl, 13.0; and 19) vitamin B-12, 0.022.

Supplemental Table S2 Name, type, dilution, and source of primary antibodies[1]

Antibody	Isotype	Dilution	Source
p300	Rabbit	1:1000	Cell Signaling Technology (Boston, MA, USA)
PGC-1α	Rabbit	1:2000	Cell Signaling Technology (Boston, MA, USA)
Sirt1	Rabbit	1:2000	Cell Signaling Technology (Boston, MA, USA)
Slow MyHC	Rabbit	1:1000	Abcam (Cambridge, MA, USA)
Fast MyHC	Rabbit	1:1000	Abcam (Cambridge, MA, USA)
ACTB	Rabbit	1:2000	Cell Signaling Technology (Boston, MA, USA)
Secondary Antibody			
HRP labelled Antibody	Goat anti rabbit	1:10,000	Santa Cruz (Santa Cruz, CA, USA)

[1]PGC-1α, peroxlsome proliferator-activated receptor-γ coactlvator-1α; Sirt1, Silent information regulator 1; Slow MyHC, slow myosin heavy chain; Fast MyHC, fast myosin heavy chain; ACTB, β-Actin.

Supplemental Table S3 Primers used for real-time quantitative PCR[1]

Gene	Primer sequence (5′–3′)	Length, bp
MyHCI	F:AAGGGCTTGAACGAGGAGTAGA<br>R:TTATTCTGCTTCCTCCAAAGGG	115
MyHCIIa	F: GCTGAGCGAGCTGAAATCC<br>R: ACTGAGACACCAGAGCTTCT	137
MyHCIIx	F: AGAAGATCAACTGAGTGAACT<br>R: AGAGCTGAGAAACTAACGTG	149
MyHCIIb	F: ATGAAGAGGAACCACATTA<br>R: TTATTGCCTCAGTAGCTTG	166
Myoglobin	F: CACCATGGGGCTCAGTGATG<br>R:CTCAGCCCTGGAAGCCTAGC	143
TnI slow	F: AGTTGAGAGGAAATCCAAGAT<br>R:CTTCAGCTTCAGGTCCTTGAT	125
TnI fast	F:TCAAGGATAAGGGCGACAGC<br>R:TGTCCATGGACGCAGCTCTT	126
18srRNA	F:CCCACGGAATCGAGAAAGAG<br>R:TTGACGGAAGGGCACCA	122

[1]MyHCI, Myosin heavy chain I; MyHCIIa, Myosin heavy chain IIa; MyHCIIx, Myosin heavy chain IIx; MyHCIIb, Myosin heavy chain IIb; TnI slow, Troponin I slow; TnI fast, Troponin I fast.

F: forward primer; R: reverse primer

Online Supplementary Material

Figure S1. Dose-response curves for C2C12 myotubes to garcinol

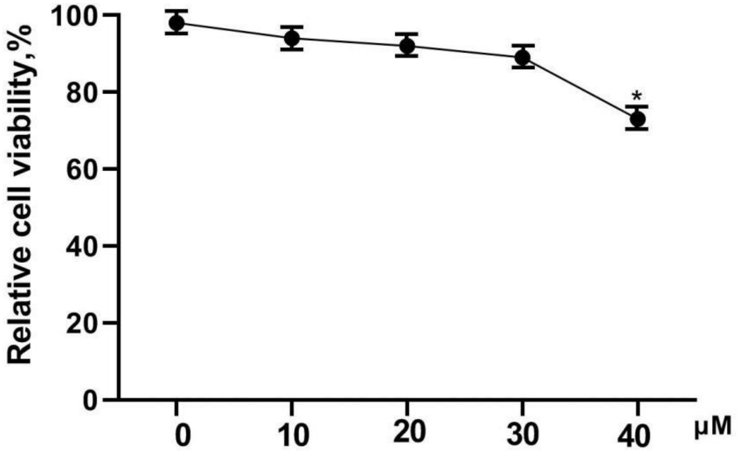

Figure S2. Time-response curve of C2C12 myotubes to garcinol

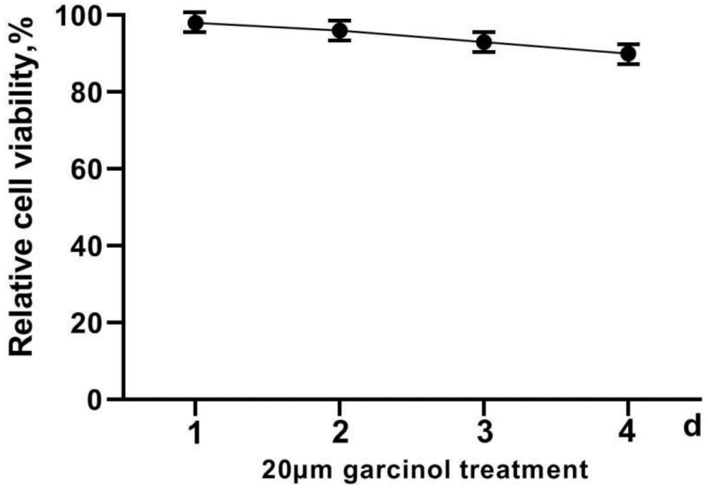

Supplement: Supplementary file 1 [file ijms-24-02702-s001.zip › ijms-2090129-supplementary.pdf]
